# Surgical treatment of the patient with locally advanced esophageal cancer and concurrent thymoma after neoadjuvant therapy: a case report

**DOI:** 10.3389/fimmu.2026.1709595

**Published:** 2026-02-26

**Authors:** Qun-Xian Zhang, Hui Liu, Juan Xu, Tao Zeng, Jun Zhang, Xiao-Fei Ren, Dan Li, Qiang Guo, Tao Liu

**Affiliations:** 1 Department of Cardiothoracic Surgery, Taihe Hospital, Hubei University of Medicine, Shiyan, China; 2 Department of Ultrasound Medicine, Taihe Hospital, Hubei University of Medicine, Shiyan, China; 3 Operating Room, Taihe Hospital, Hubei University of Medicine, Shiyan, China; 4 Department of Oncology, Taihe Hospital, Hubei University of Medicine, Shiyan, China

**Keywords:** cancer nodules, esophageal squamous cell carcinoma, lymph node metastasis, neoadjuvant therapy, thymoma

## Abstract

Locally advanced esophageal squamous cell carcinoma (ESCC) can be treated effectively with surgical resection following downstaging *via* preoperative neoadjuvant therapy. This case report details the successful management of a patient with ESCC complicated by thymoma, who underwent one cycle of neoadjuvant therapy followed by surgery, resulting in improved survival. The patient initially presented with dysphagia and was diagnosed with ESCC at a local hospital, where gastroscopy revealed a neoplasm located 28–30 cm from the incisors. Pathological examination confirmed ESCC, and the clinical stage was cT2N2M0, stage III. The patient received chemotherapy (paclitaxel + cisplatin) combined with toripalimab at the local hospital but developed elevated thyroid hormone levels during treatment. Post-treatment enhanced chest CT scans at our institution showed a malignant tumor in the mid-to-lower esophagus, enlarged mediastinal lymph nodes, and an anterior mediastinal mass suggestive of thymoma, along with emphysema. Barium meal radiography indicated mild stiffness but good dilatation of the esophageal wall, with a normal cardia opening. Endoscopic ultrasound revealed ESCC invading the muscularis propria, chronic atrophic gastritis with erosion, and gastric xanthoma. Following multidisciplinary team (MDT) evaluation, the patient proceeded to thoracoscopic radical esophagectomy and resection of the mediastinal mass. Postoperative pathology showed ypT1bN2M0, stage IIIB, poorly differentiated ESCC with necrosis, measuring 2.5 × 1.6 × 0.4 cm, and invading the submucosa without vascular tumor thrombus or perineural invasion. Surgical margins, including those at the gastric resection site and proximal anastomosis, were free of tumor. Metastases were identified in the right recurrent laryngeal nerve chain and perigastric lymph nodes (2/4). The anterior mediastinal mass was confirmed as thymoma. Postoperative management included oxygen support, nebulization, analgesia, gastrointestinal decompression, antibiotics (piperacillin/cefradine), expectorants, antispasmodics, acid suppression, intravenous and enteral nutrition, and albumin supplementation. The patient recovered well and was discharged on postoperative day 12. Chemotherapy and radiotherapy were administered at the local hospital. During the 10-month follow-up, the patient remained recurrence-free and reported no dysphagia. This case provides valuable insights and therapeutic strategies for managing similar cases.

## Background

**Figure 1:**
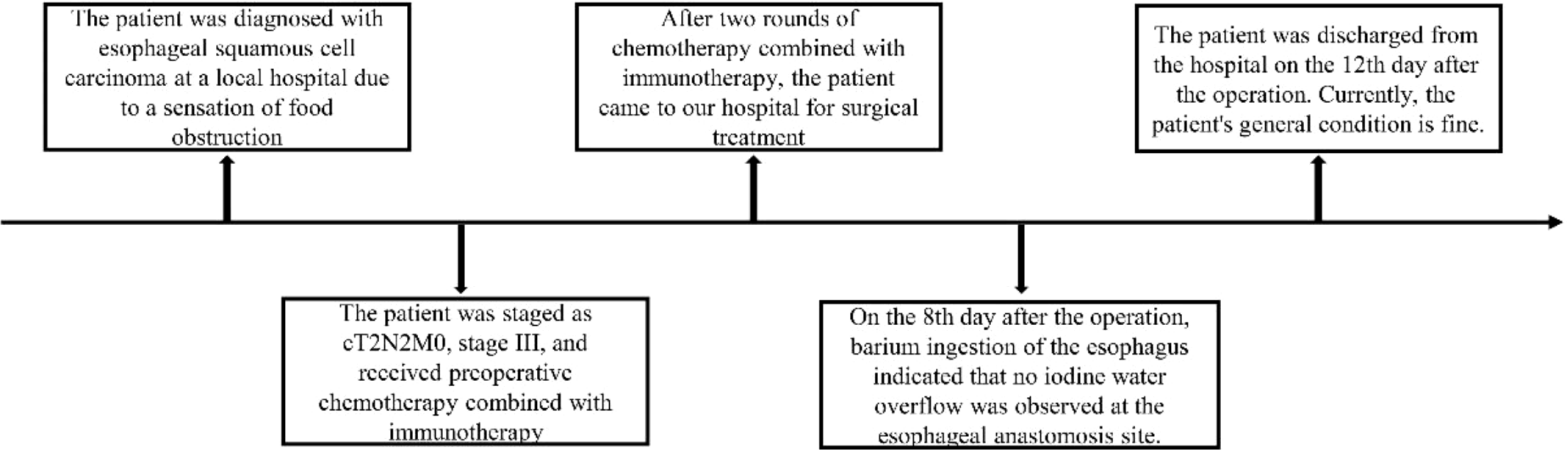
Time flow chart of the patient’s treatment.

Esophageal cancer is a common malignancy, with esophageal squamous cell carcinoma (ESCC) being one of the most prevalent subtypes in China. In recent years, neoadjuvant therapy followed by surgical resection has significantly improved survival in selected patients with locally advanced ESCC, marking a promising advancement in treatment. While current studies document successful surgical outcomes after neoadjuvant therapy in patients with isolated locally advanced ESCC, cases involving concurrent ESCC and thymoma remain rare. This report presents a patient with ESCC complicated by thymoma who became eligible for surgery after preoperative neoadjuvant therapy. The procedure was successfully completed without postoperative anastomotic leakage (**Figure 1**), leading to prolonged survival and improved quality of life. This case shares clinical experience and offers insights for treatment strategies in similar complex cases.

## Case description

The patient, a 58-year-old male, presented to Zhushan People’s Hospital with difficulty swallowing dry and hard foods. Electronic gastroscopy revealed a neoplasm located 28–30 cm from the incisors in the esophagus. Additional findings included multiple erosions and diffuse redness in the gastric fundus and body, along with congested and edematous antral mucosa. Helicobacter pylori infection was detected, consistent with infectious gastritis. Pathological examination confirmed ESCC. Chest and upper abdominal CT scans indicated a space-occupying lesion in the anterior mediastinum and thickening of the esophageal wall. Based on the clinical presentation and diagnostic investigations, the patient was diagnosed with stage III esophageal cancer, classified as cT2N2M0. After discussions with the patient and his family, one cycle of neoadjuvant chemotherapy (paclitaxel + cisplatin) combined with toripalimab was administered. Following treatment, the patient developed elevated thyroid hormone levels and experienced reduced energy and appetite. After an endocrinology consultation, the patient was diagnosed with severe hyperthyroidism and treated with prednisone and beta-blockers. With appropriate management, the patient’s thyroid hormone levels returned to normal. After a thorough evaluation, the patient and his family decided to pursue further treatment at our hospital.

Upon admission, the patient’s vital signs were as follows: temperature 36.5 °C, pulse 82 beats per minute, respiratory rate 20 breaths per minute, and blood pressure 111/72 mmHg. Breath sounds were clear bilaterally, with no dry or wet rales. Cardiac auscultation revealed no pathological murmurs. The abdomen was flat and soft, with no tenderness or rebound tenderness. The liver and spleen were not palpable below the costal margin, and there was no edema in the lower extremities.

**Figure 2:**
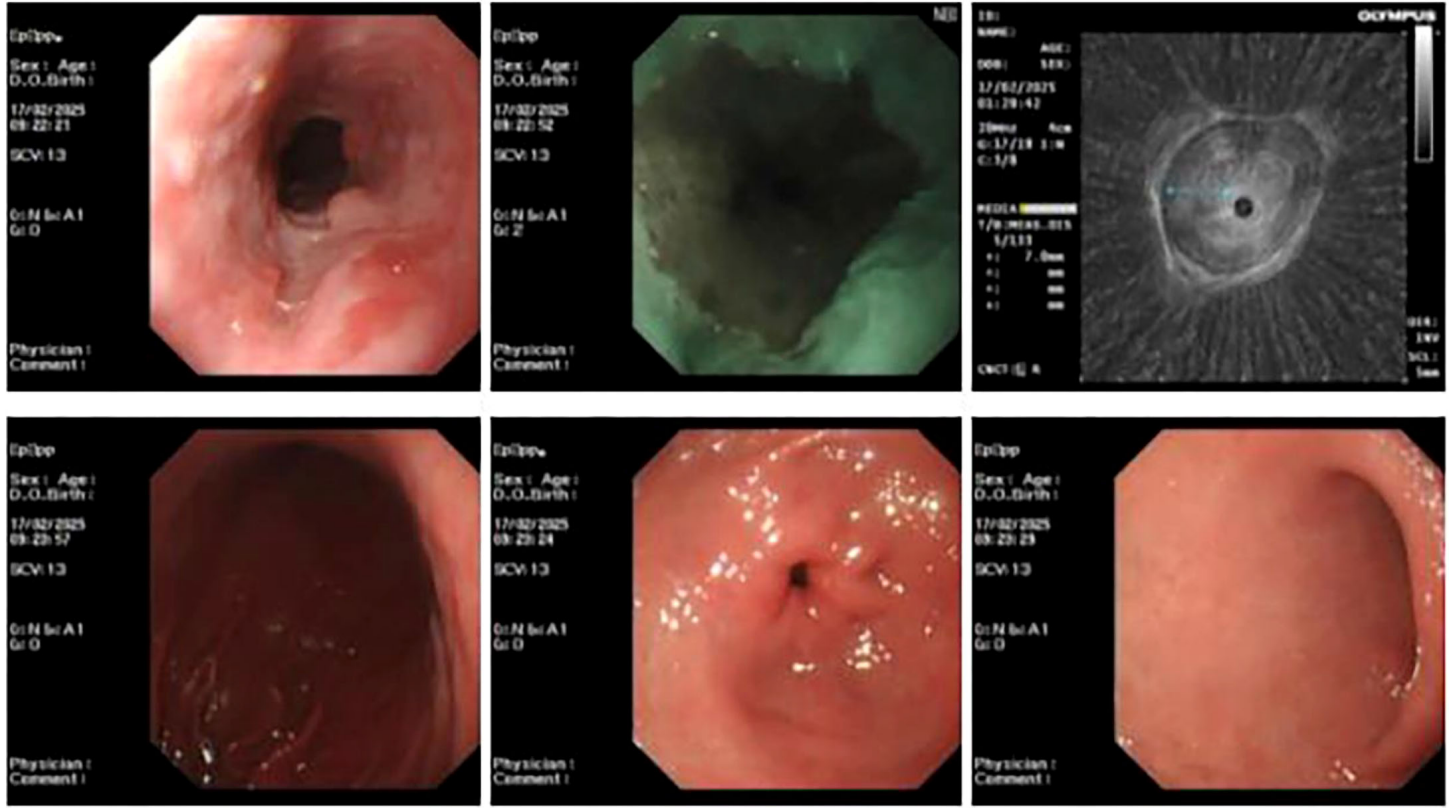
Ultrasound gastroscopy showing esophageal lesions at our hospital.

**Figure 3:**
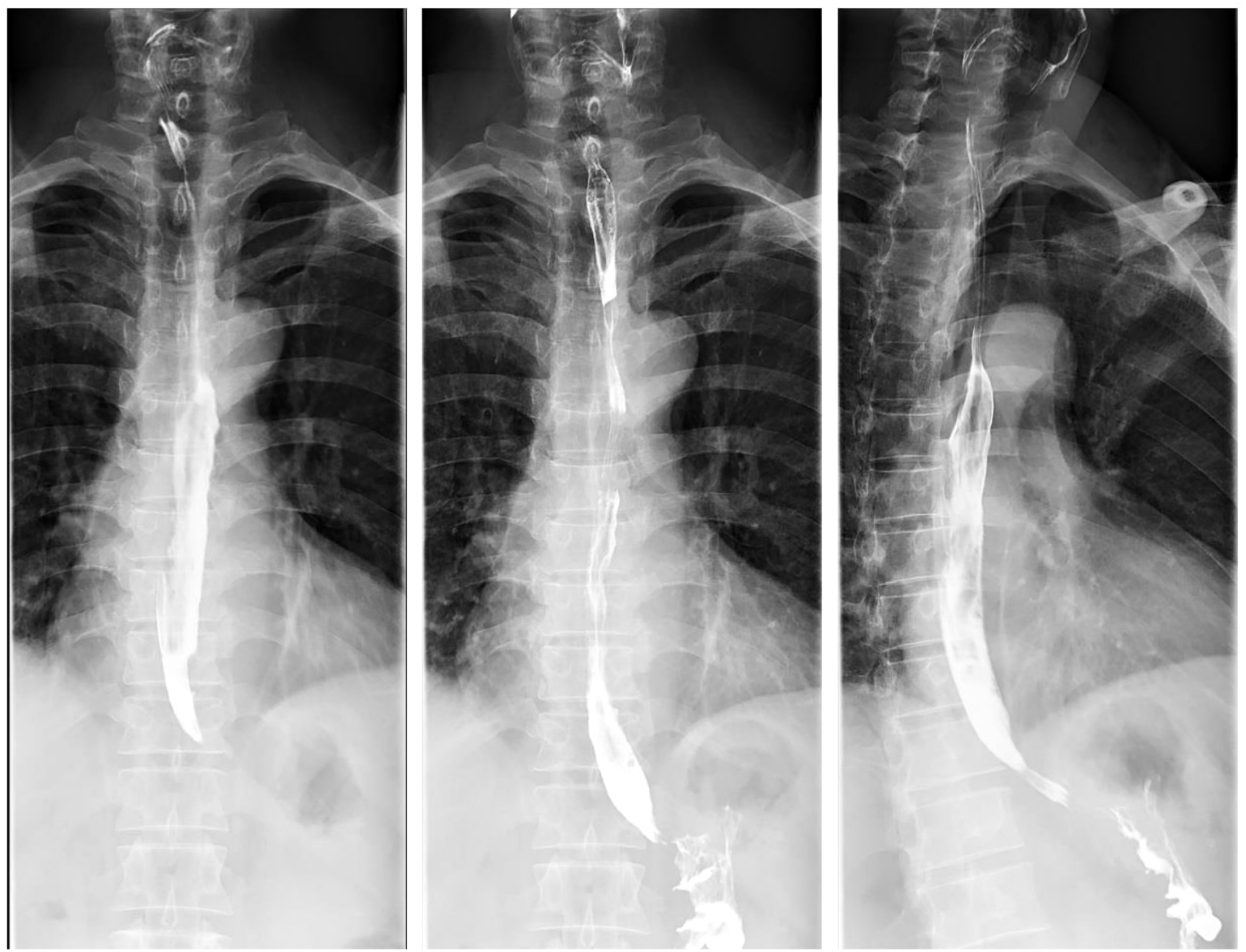
Barium meal radiography of the esophagus showing esophageal lesions at our hospital.

**Figure 4:**
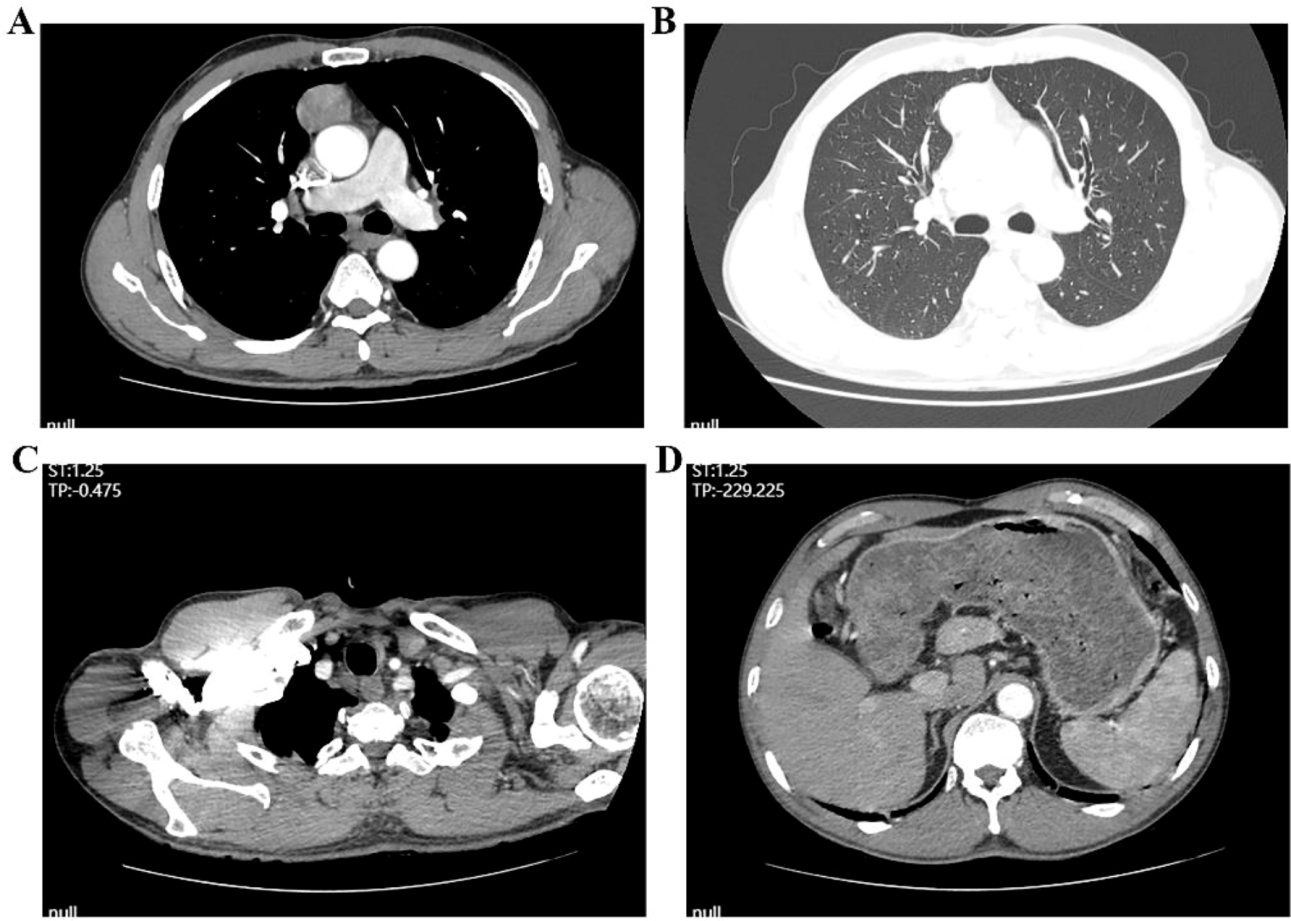
Enhanced chest CT showing esophageal lesions and lymph node metastasis. **(A, B)** Mediastinal windowing reveals a soft tissue mass within the mediastinum; **(C, D)** Serial images demonstrate evidence of mediastinal lymphadenopathy.

**Figure 5:**
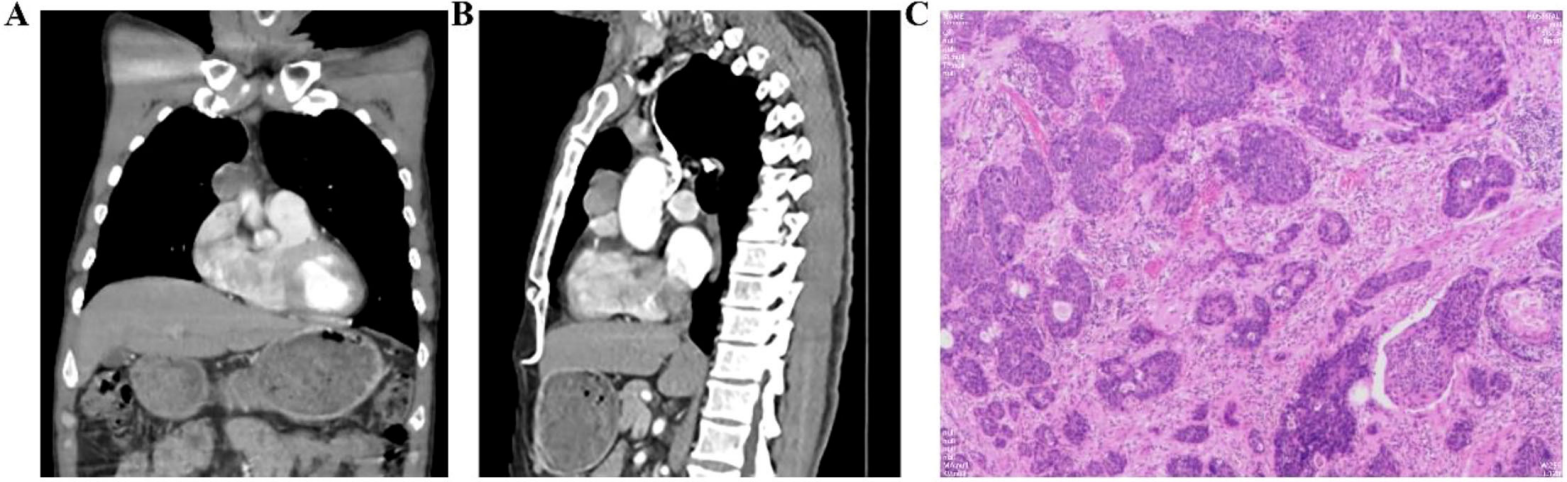
Enhanced chest CT showing mediastinal masses and postoperative pathological results of the esophagus. **(A, B)** mediastinal masses; **(C)** The pathological result of the esophagus.

Endoscopic ultrasound at our hospital revealed an irregular bulge in the esophageal mucosa located 27–35 cm from the incisors, involving the muscularis propria, with wall thickening measuring approximately 0.7 cm. Chronic atrophic gastritis with erosion was also observed (**Figure 2**). Pathological examination confirmed ESCC. Barium meal radiography showed mild stiffness but good dilatation of the mid-to-lower esophageal wall after chemotherapy, with no obvious niche or filling defect. The remaining esophageal segments appeared normal, and the cardia opened well (**Figure 3**). Enhanced chest CT indicated a probable malignant tumor in the mid-to-lower esophagus, with enlarged mediastinal lymph nodes. A space-occupying lesion approximately 3.8 × 2.5 cm was observed in the anterior mediastinum, suggestive of a tumor. Additional findings included scattered micronodules in both lungs and pleura, fibrous foci in the right middle lobe and left lower lobe, and emphysema (**Figures 4**, **5A,B**). After completing the preoperative assessments, the patient underwent thoracoscopic radical resection of ESCC, thoracoscopic resection of the mediastinal lesion, and release of thoracic adhesions on March 3, 2025.

Surgical procedure: General anesthesia and intubation were successful. The patient was positioned in the left supine position, and routine disinfection and draping were performed. Incisions of approximately 0.5 to 1.5 cm were made at the 3rd and 5th intercostal spaces at the right axillary front line, the 7th intercostal space at the middle axillary line, and the 8th intercostal space at the posterior axillary line. Artificial pneumothorax was induced to collapse the lung tissue. No effusion was observed in the thoracic cavity, although there was extensive adhesion present. The adhesion bands were released, and exploration revealed that the anterior mediastinal tumor was located at the lower pole of the thymus and the upper edge of the pericardium, measuring approximately 4 × 3 cm. The boundaries of the tumor with the pleura, thymus, pericardium, ascending aorta, and anterior chest wall were unclear, with surrounding tissues exhibiting fibrous tissue hyperplasia. The fibrous tissue around the tumor was separated using an ultrasound knife and electrocoagulation. After resecting the adipose tissue in front of the pericardium and at the angle of the cardiodiaphragm, the mediastinal tumor was excised, and its nourishing blood vessels were ligated with Hemlock. The tumor was located in the middle and lower segments of the esophagus, extending outward. The intrathoracic esophagus was mobilized using an electrohook combined with an ultrasonic scalpel, and multiple lymph node groups, including the right and left recurrent laryngeal nerve chains, subcarinal lymph nodes, and para-esophageal lymph nodes, were dissected. Following complete hemostasis, a thoracic drainage tube was placed at the 7th intercostal space along the midaxillary line. The patient was then repositioned to the supine position. After routine disinfection and draping, incisions of 0.5–1.5 cm were made under the xiphoid process, at the umbilicus, on both sides of the umbilicus, and on the right abdomen to establish the laparoscopic approach. Upon creating artificial pneumoperitoneum, extensive adhesions in the abdominal cavity were encountered, and adhesion release surgery was performed. The gastric and esophageal hiatus was freed using an ultrasonic scalpel, the short gastric artery was treated, and the left gastric artery was clamped with a Hemolok clip and subsequently divided. Left gastric lymph nodes were cleared. A 6-cm-long incision was made along the anterior edge of the sternocleidomastoid muscle on the left side of the neck, and the incision was gradually deepened to expose the esophageal space. The esophagus was pulled out through the incision, severed, and the distal end was tethered with a gastric tube for traction. After removal of the laparoscope, the incision under the xiphoid process was enlarged, and the stomach and esophagus were extracted to form a tubular stomach. The tubular stomach was then pulled through the cervical incision using the traction gastric tube. Four intermittent sutures were placed at the posterior wall of the esophagus and the greater curvature of the tubular stomach to complete the anastomosis of the third layer of the posterior wall. The esophagus and tubular stomach were secured with trilateral forceps. The esophageal stump was pruned, and the muscular and mucosal layers were separated. A 2-cm diameter anastomosis was made at an appropriate position in the tubular stomach. The esophagogastric cervical anastomosis was performed with a combination of intermittent and continuous sutures using 4–0 antibacterial Michio thread. The operation was completed after adjusting the gastric tube and duodenal nutrition tube to the proper positions.

Postoperative management included symptomatic and supportive care, consisting of oxygen support, nebulization, analgesia, gastrointestinal decompression, antibiotic therapy (piperacillin/cefradine), expectorants, antispasmodics, acid suppression, intravenous and enteral nutrition, albumin supplementation, and therapeutic fiberoptic bronchoscopy with bronchoalveolar lavage. Pathological examination of the resected specimen confirmed ypT1bN2M0, stage IIIB ESCC (**Figure 5C**). The tumor measured 2.5 × 1.6 × 0.4 cm, was poorly differentiated with necrotic areas, and invaded the submucosa. No lymphovascular invasion or perineural involvement was observed. The adjacent gastric mucosa exhibited moderate chronic inflammation with intestinal metaplasia. All surgical margins, including the gastric resection margin and proximal esophageal stump, were tumor-free. Lymph node involvement was identified in one node of the right recurrent laryngeal nerve chain and two of four pericardial lymph nodes. No metastasis was detected in the paraspinal upper esophageal (0/4), subcarinal (0/7), left recurrent laryngeal nerve chain (0/1), or left gastric lymph nodes (0/12). The anterior mediastinal mass was confirmed as a type A thymoma, measuring 4.6×4.3×2 cm and classified as Masaoka stage I.

**Figure 6:**
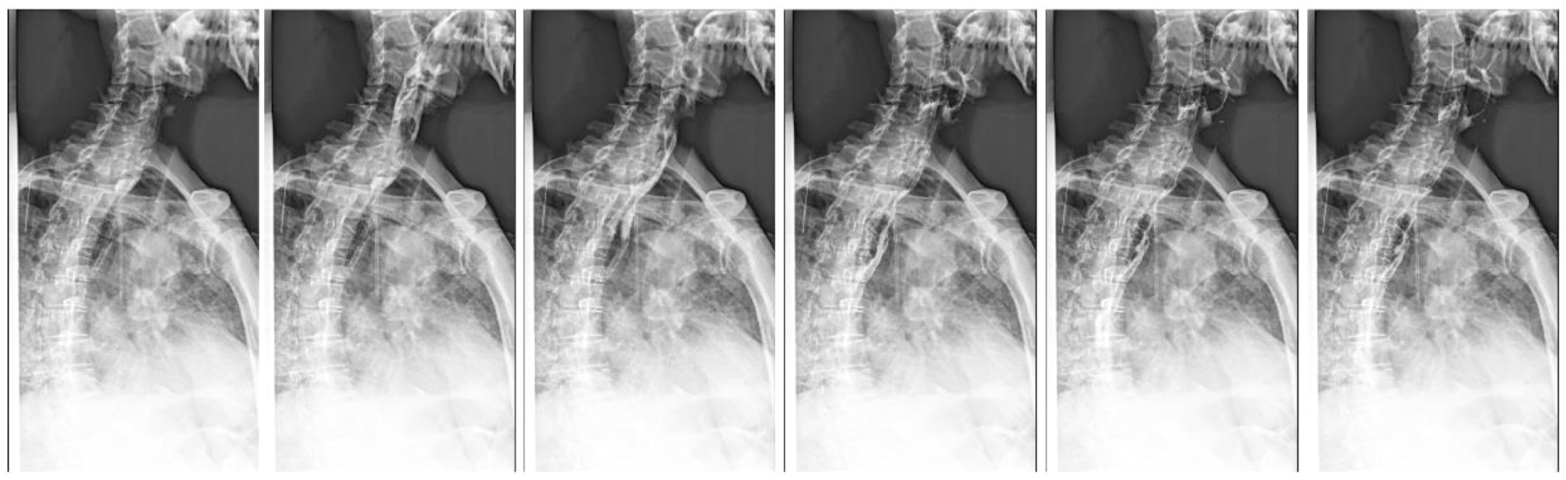
Esophageal iodine contrast study illustrating the condition of the esophageal anastomosis.

**Figure 7:**
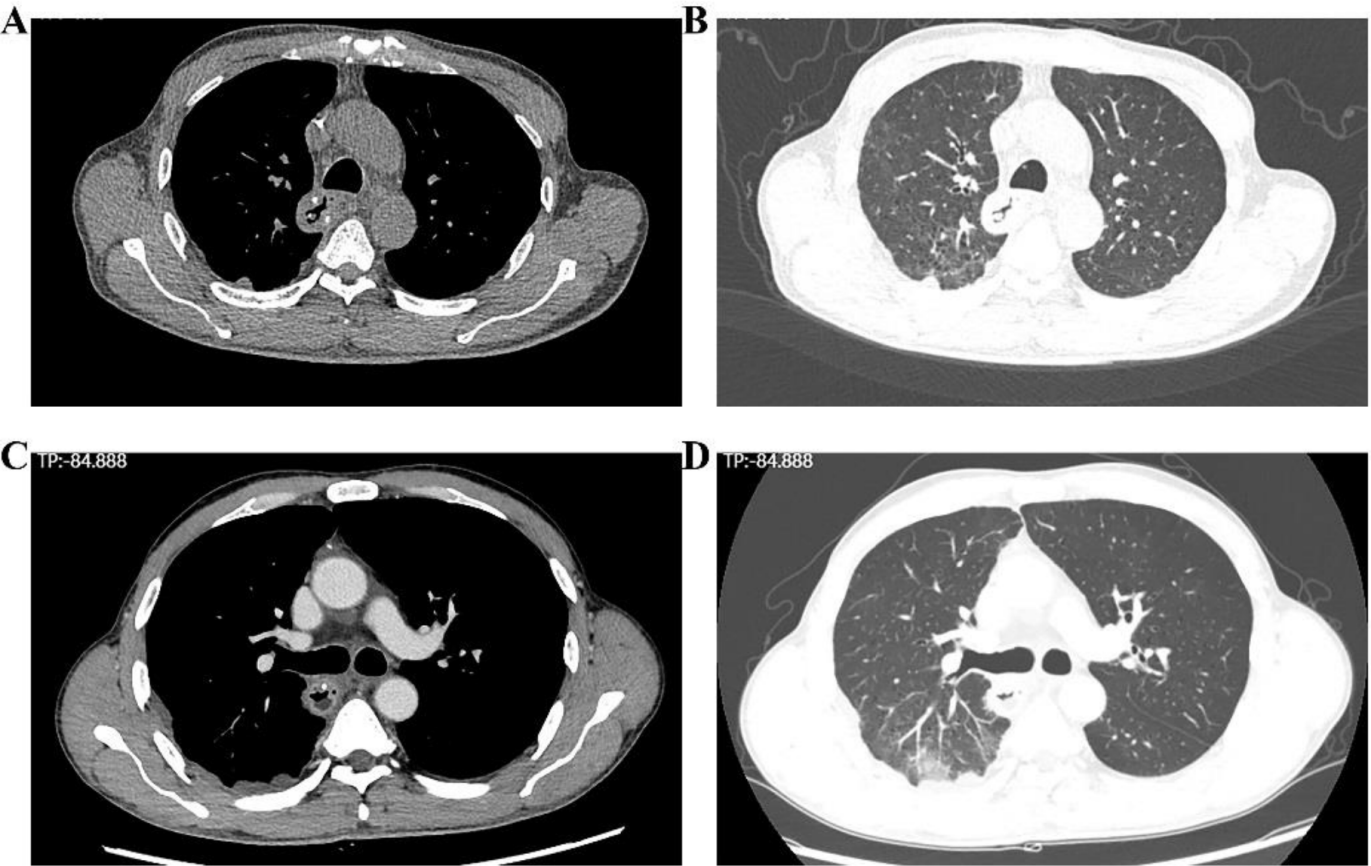
Chest CT scan showing postoperative changes in the esophagus. **(A, B)** The results of the chest CT scan of the patient before discharge; **(C, D)** The results of the chest CT scan of the patient after discharge.

On postoperative day 8, an iodine-based contrast study showed mild anastomotic stenosis (approximately 0.8 cm in diameter) without evidence of contrast extravasation (**Figure 6**). The patient remained asymptomatic, tolerated a liquid diet, and showed no signs of fever or other discomfort. Chest CT on postoperative day 11 revealed typical postoperative changes following esophagectomy, reduced bilateral pulmonary inflammation, emphysema, segmental atelectasis in the right middle lobe and left upper lobe, and a small bilateral pleural effusion (**Figures 7A, B**). The patient was discharged 12 days after the surgery and made a full recovery. Follow-up contrast-enhanced chest CT performed more than one month post-surgery showed partial pulmonary expansion, persistent emphysema, and minimal residual bilateral pleural effusion (**Figures 7C, D**). The patient continued postoperative chemotherapy and radiotherapy at the local hospital. During the 10-month follow-up, the patient remained free of recurrence, no dysphagia, and experienced slight weight loss, as noted during a telephone consultation.

## Discussion

Esophageal cancer has one of the highest incidences and mortality rates among malignant tumors (1), with most patients presenting due to dysphagia. Once diagnosed with locally advanced ESCC, the 5-year survival rate typically does not exceed 20% (1). Neoadjuvant therapy has shown promising outcomes in this patient population. In this case, a patient diagnosed with locally advanced ESCC *via* gastroscopic biopsy successfully underwent surgery after chemotherapy combined with immunotherapy, with no postoperative complications and smooth recovery, aligning with current literature reports.

Several cases have documented concurrent surgical management for esophageal cancer with other diseases (2). For example, Zhang et al. reported successful simultaneous resection of early esophageal cancer and hepatogastric schwannoma (2). However, no prior studies have described cases of ESCC complicated by thymoma or involving neoadjuvant immunotherapy plus chemotherapy followed by surgery. This case represents the first documented instance of a patient undergoing thoracoscopic radical esophagectomy combined with thoracoscopic resection of a mediastinal lesion and lysis of thoracic adhesions. Postoperative supportive care included oxygen therapy, nebulization, analgesia, gastrointestinal decompression, antibiotics, expectorants, antispasmodics, acid suppression, intravenous and enteral nutrition, albumin supplementation, and fiberoptic bronchoscopy with bronchoalveolar lavage. Pathological analysis confirmed poorly differentiated ESCC with necrosis and submucosal invasion. Metastases were found in one lymph node along the right recurrent laryngeal nerve chain and in two of four pericardial lymph nodes. The anterior mediastinal mass was identified as a type A thymoma. The patient was discharged in good condition.

Neoadjuvant immunotherapy can induce immune-related adverse events (irAEs) affecting organs such as the thyroid, skin, liver, and lungs, potentially requiring early discontinuation of treatment (3, 4). In this case, although irAEs occurred, they were effectively managed with appropriate intervention, enabling the patient to proceed to surgery. It is important to note that the neoadjuvant immunotherapy and chemotherapy were administered at a local hospital rather than our institution. Several limitations exist, including incomplete preoperative imaging data such as contrast-enhanced chest CT, PD-L1 expression, and the detailed treatment process, which hindered a comprehensive evaluation of the treatment response prior to surgery. Additionally, most thymomas exhibit elevated PD-L1 expression, making them a viable target for immune checkpoint inhibitors (5, 6). Moreover, the thymus plays a central role in T-cell development and selection. The frequent association of thymoma with autoimmune diseases suggests a pre-existing dysregulated immune state in these patients. This inherent immune dysregulation may not only enhance tumor sensitivity to immunotherapy but also contribute to the heightened susceptibility to severe irAEs.

Overall, a triple-incision radical esophagectomy combined with thymoma resection was successfully performed, with no anastomotic leakage or major complications. The patient subsequently received adjuvant therapy at the local hospital. Telephone follow-up indicates that the patient remains in good general condition, with no evidence of distant recurrence or metastasis. In conclusion, surgical intervention should be considered whenever feasible in patients with esophageal cancer, particularly those with concurrent diseases, to reduce tumor burden and minimize the need for multiple operations. This case provides valuable insights and practical strategies for clinicians managing similar complex cases.

## Data Availability

The original contributions presented in the study are included in the article/supplementary material. Further inquiries can be directed to the corresponding authors.
